# Endoscopic Resection of Nasal Mucosal Melanoma Using Temporary Transseptal Access

**DOI:** 10.7759/cureus.26904

**Published:** 2022-07-15

**Authors:** Jun Suzuki, Kenjiro Higashi, Tomotaka Hemmi, Hiroyuki Ikushima, Yukio Katori

**Affiliations:** 1 Department of Otolaryngology Head and Neck Surgery, Tohoku University School of Medicine, Sendai, JPN

**Keywords:** inferior turbinate, transseptal access, endoscopic resection, nasal cavity, mucosal melanoma

## Abstract

Nasal mucosal melanoma (NMM) is a rare tumor with a poor prognosis. Although an endoscopic resection of malignant nasal tumor now becomes a treatment of choice when the surgical margin can be secured, it is still controversial. We report three cases of NMM that was successfully removed en bloc with clear margins by endoscopic surgery with temporal transseptal access. Cases of a 78-year-old woman, an 83-year-old man, and an 81-year-old man with NMM arising from the inferior turbinate who underwent endoscopic resection of the tumor are discussed in detail. We made temporal transseptal access using septal mucosal flaps. We moved the nasal cavity-occupying tumor to the contralateral side to create a working space to perform endoscopic en bloc resections. This technique is simple yet effective and less invasive than open craniofacial resections for removing malignancies arising from the inferior turbinate.

## Introduction

Nasal mucosal melanoma (NMM) is a rare and highly malignant tumor with a poor prognosis [1]. Surgical treatments, such as an open craniofacial resection, which allow en bloc resection with clear margins, are the primary treatment modalities in resectable cases. Its incidence is slightly higher in individuals over 60 years of age than those below that age [1]. When NMM occurs in individuals in the middle-old or oldest-old age brackets, a highly invasive surgery must be carefully considered. This is due to a high locoregional recurrence, high distant metastasis rates after complete resection, and low five-year overall survival rates of about 20-40% [2-5].

Over the past two decades, the indication for endoscopic surgery for malignant tumors in the nasal cavity has expanded. Some studies have reported that endoscopic resection is comparable to open approaches in correctly selected patients [6, 7]. For tumors that fill the nasal cavity or have a pedicle that is difficult to identify, a layered piecemeal resection with a continuous evaluation of the surgical margins has been proposed and has become widespread [6]. However, piecemeal resection has a risk of dissemination, and its adaption to NMM treatment is prudent. In addition, recent studies have suggested that transseptal access could increase the mobility of benign sinonasal tumors and allow en bloc resection [8, 9]. Considering these issues, we have come up with the idea of applying a transseptal approach to the resection of malignant tumors. We recently had three cases where we performed an endoscopic en bloc resection of an NMM from the inferior turbinate using temporary transseptal access and endoscopic surgery. In this case report, we present an effective and less invasive technique to remove malignancy arising from the inferior turbinate of the nasal cavity.

## Case presentation

Case 1

**Figure 1:**
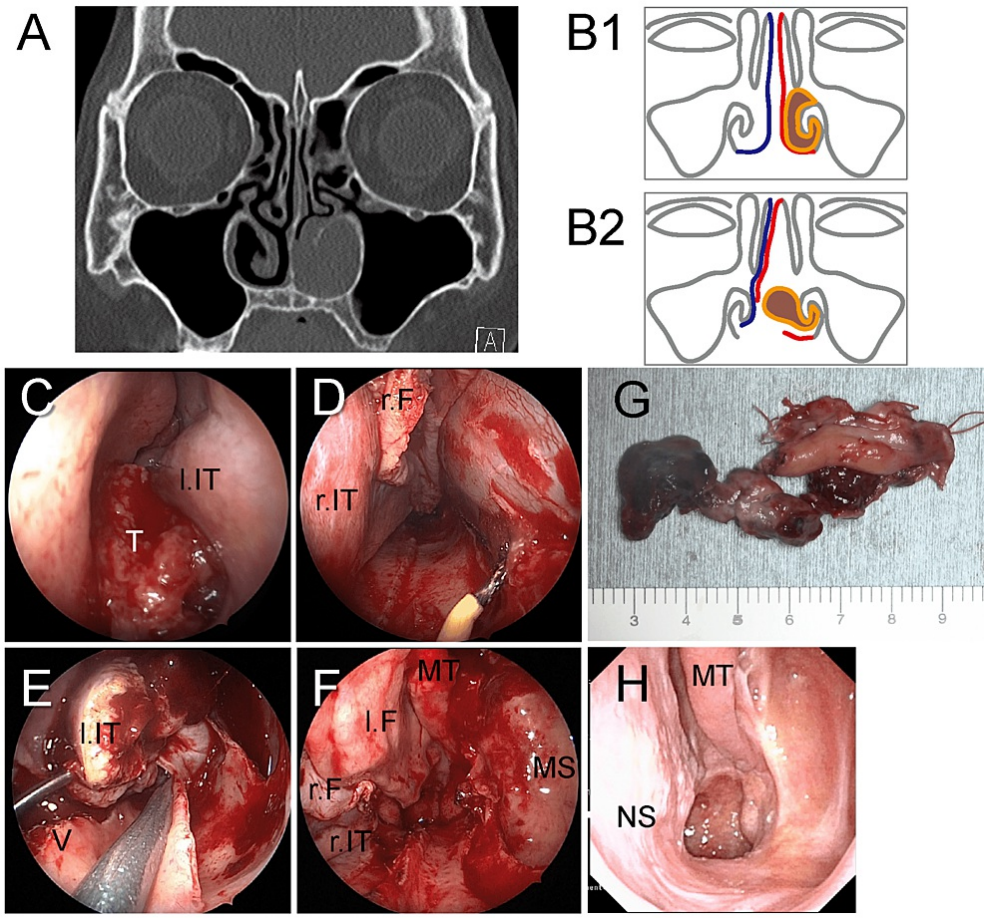
Case 1. A: Preoperative CT showing a nasal cavity-occupying tumor without bone destruction; B: Schematic illustration of the transseptal access (B1: Before the procedure; B2: After the procedure; Blue: Septum mucosa of the intact side; Red: Septum mucosa of the tumor side; Brown: Tumor); C: Preoperative endoscopic image of the tumor; D: Horizontal incision of the septal mucosa of the tumor side; E: Retraction of the tumor through the transseptal access to visualize clear surgical margins; F: Left nasal cavity after endoscopic medial maxillectomy; G: A gross image of the resected tumor; H: Endoscopic view of the left nasal cavity at 15 months after the first surgery; T: Tumor; l.IT: Left inferior turbinate; r.IT: Right inferior turbinate; r.F: Right septal mucosa flap; V: Vomer; MT: Middle turbinate; MS: Maxillary sinus; NS: Nasal septum.

The patient was a 78-year-old female who had been experiencing left nasal bleeding for seven months. An endoscopic examination showed an easy-bleeding tumor occupying the lower part of the left nasal cavity. CT and MRI revealed that the tumor arose from the left anterior inferior turbinate without erosion or bone destruction (Figure 1A). An incisional biopsy was performed, and the histology revealed an NMM. Additional examinations, including enhanced neck and chest CT scans and a positron emission tomography (PET), revealed no metastasis, resulting in the diagnosis of clinical T3N0M0 (stage III).

Endoscopic resection was performed under general anesthesia (Figure 1A-1G). Per the Killian method, a vertical incision was made on the intact side after administration of a submucosal injection of 0.5% lidocaine and 1:2000,000 adrenaline. A nasal septoplasty was performed to remove the excess cartilage and bone. On the intact side, a vertical incision was made at the posterior end of the nasal septum, and an inferior horizontal incision was made on the nasal floor. Next, the following incisions were made: a vertical incision on the left septal mucosa 5 mm behind the right vertical incision, a horizontal incision at the height of the rest of the vomer, and a vertical incision at the posterior end of the nasal septum (Figure 1D). These incisions created space for the tumor to be laterally displaced over the midline (Figure 1B). Next, we performed endoscopic medial maxillectomy (EMM) for en bloc resection of the tumor arising from the inferior turbinate. The transseptal access enabled us to manually retract the tumor and visualize the margins (Figure 1E-1F). The septal mucosa was reconstructed by medially replacing both mucosal flaps and using a suture for each vertical incision. A fibrin glue stabilized the flaps to the nasal floor. Histological examination suggested that the tumor was an amelanotic melanoma with negative surgical margins. Postoperatively, no septal perforation, facial disfigurement, cheek numbness, or mastication disorder occurred. Local recurrence occurred on the nasal floor nine months after the first surgery, and salvage endoscopic surgery was performed. The patient remains cancer-free without complications, as mentioned earlier, 21 months after the first surgery (Figure 1H).

Case 2

**Figure 2:**
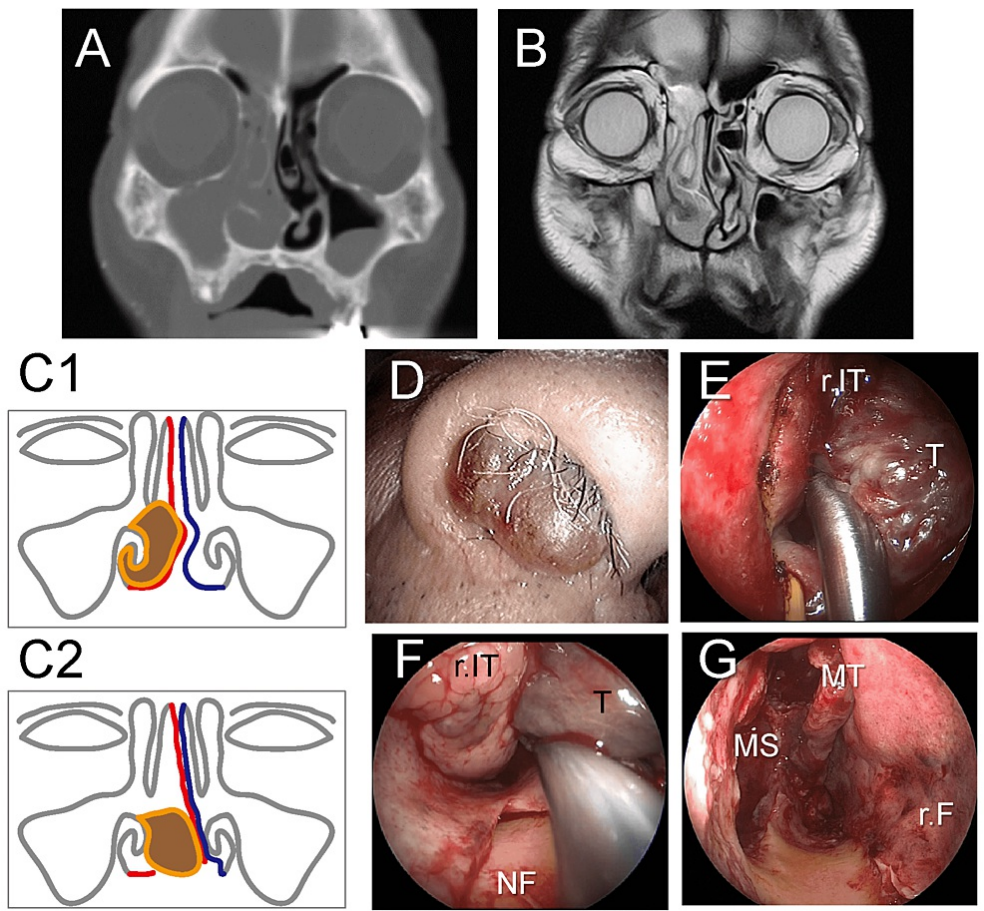
Case 2. A-B: Preoperative CT (A) and MRI (B) showing a nasal cavity-occupying tumor and maxillary and ethmoidal sinusitis; C: Schematic illustration of the transseptal access (C1: Before the procedure; C2: After the procedure; Blue: Septum mucosa of the intact side; Red: Septum mucosa of the tumor side; Brown: Tumor); D: Preoperative image of the tumor; E: Vertical incision of the lateral nasal wall for endoscopic medial maxillectomy after displacing the tumor to the intact side; F: Retraction of the tumor through the transseptal access to visualize clear surgical margins; G: Right nasal cavity after endoscopic medial maxillectomy; r.IT: Right inferior turbinate; T: Tumor; NF: Nasal floor; r.F: Right septal mucosa flap; MT: Middle turbinate; MS: Maxillary sinus.

The patient was an 83-year-old male who had been experiencing right nasal bleeding for three months. Nasal examination showed a large tumor protruding from the anterior nasal cavity. CT scan and MRI revealed a tumor in the right inferior turbinate without erosion or bone destruction and septal deviation to the left (Figure 2A-2B). An incisional biopsy was performed, and histology revealed an NMM. Additional examinations revealed no metastasis findings, resulting in the diagnosis of clinical T3N0M0 (stage III). Endoscopic resection was executed using the same procedure as that in Case 1 with a modification (Figure 2C-2G). After the submucosal injection, a vertical incision was made on the left side of the nasal septum. Next, a nasal septoplasty was performed, and the left mucosal flap was raised to the posterior end of the nasal septum and the lateral end of the nasal floor. Next, a vertical incision on the right septal mucosa was made 5 mm ahead of the left vertical incision, a horizontal incision at the nasal floor, and a vertical incision at the posterior end of the nasal septum. Using extensive transseptal access (Figure 2C), we pushed the protruding tumor to the left nasal cavity, performed an EMM, and completed an en bloc resection (Figure 2D-2G). Histological examination suggested that the tumor was an amelanotic melanoma with negative surgical margins. Although a pin-hole size septal perforation without any symptoms occurred, there was no facial disfigurement, cheek numbness, or mastication disorder. His quality of life was maintained. Local recurrence, cervical lymph node metastasis, and pelvic metastasis occurred eight months after the first surgery, and palliative radiation therapy was performed. Unfortunately, the patient died 17 months after the first surgery due to distant metastasis.

Case 3

**Figure 3:**
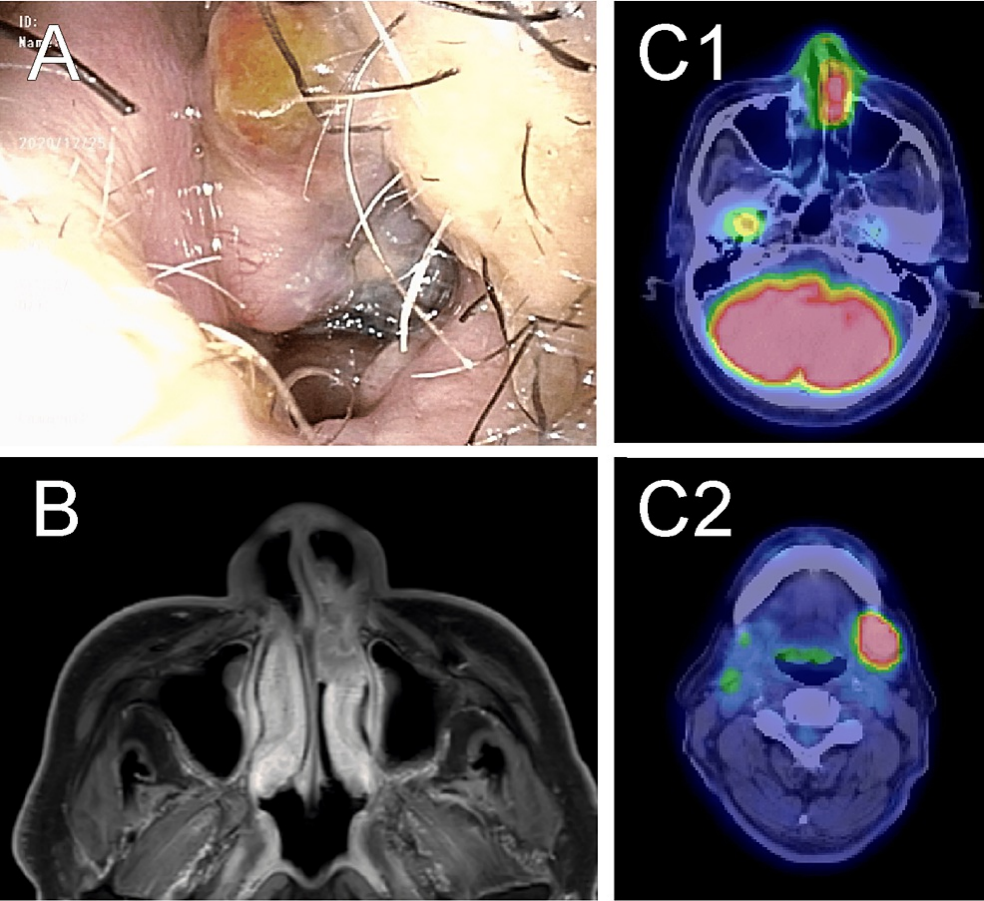
Case 3. A: Preoperative image of the tumor; B: Preoperative MRI showing a nasal cavity-occupying tumor; C: Positron emission tomography/CT images showing intense uptakes of 18F-FDG in the left nasal cavity (C1) and the left cervical lymph node (C2).

The patient was an 81-year-old male who had been experiencing left nasal obstruction for two months. An endoscopic examination showed an irregular tumor occupying the common nasal meatus of the left nasal cavity (Figure 3A). MRI revealed that the tumor arose from the left anterior inferior turbinate and the nasal vestibule (Figure 3B). PET showed the presence of the left neck lymph node metastasis (Figure 3C). After diagnosing an NMM in clinical T3N1M0 (stage IVa), we performed similar en bloc tumor resections using temporal transseptal access with negative surgical margins and left modified radical neck dissection under general anesthesia. Histological examination suggested that the tumor was an amelanotic melanoma with negative surgical margins. Postoperatively, no septal perforation, facial disfigurement, cheek numbness, or mastication disorder occurred. Right cervical lymph node recurrence occurred eight months after the first surgery, and salvage right modified radical neck dissection was performed. The patient remains cancer-free 14 months after the first surgery without the complications mentioned earlier. A summary of three cases is shown in Table 1. Informed consent has been obtained from all patients included in this study.

**Table 1:** Summary of three cases of nasal malignant melanoma treated with endoscopic surgery with temporal transseptal access. L: Left; R: Right; IT: Inferior turbinate; NV: Nasal vestibule; LN: Lymph node; ES: Endoscopic surgery; RT: Radiation therapy; ND: Neck dissection.

Case	Age	Sex	Side	Location	T	N	M	Stage	Neck dissection	Recurrence	Time to recurrence (months)	Salvage treatment	Total follow-up (months)	Outcome
1	78	Female	L	IT	3	0	0	3	-	Local	10	ES	21	Disease-free survival
2	83	Male	R	IT	3	0	0	3	-	Local, LN, Bone	8	RT	17	Death
3	81	Male	L	IT, NV	4a	1	0	4a	L	LN	8	R. ND	14	Disease-free survival

## Discussion

In this report, we described three cases of rare NMMs that were successfully removed using en bloc resection with negative margins using temporal transseptal access. This technique is simple and less invasive compared with open approaches; therefore, we recommend it as a feasible treatment modality for resectable NMM.

Owing to the rarity of NMM, it is difficult to recruit a large enough cohort to compare endoscopic resection with the established open approaches [10]. Considering the poor prognosis of NMM with high local recurrence and distant metastasis rates, a European position paper mentions that whether the tumor is removed endoscopically via an external incision or midfacial degloving appears to make little difference to the prognosis of NMM [11]. Moreover, recent research suggests that endoscopic resection has better survival outcomes than open surgery [12, 13]. Although endoscopic resection, especially endoscopic piecemeal resection, has a perceived risk of tumor implantation owing to tumor fragmentation, past studies indicated that the results of endoscopic resection are not worse than those of open approaches [14, 15]. Therefore, although there is limited evidence indicating noninferiority of the endoscopic approach to open approaches for resection of NMM, it must be acceptable to perform endoscopic surgeries in patients with NMM in which surgical margins are expected to be available, especially in the elderly who cannot tolerate highly invasive surgeries. Although the number of cases and observation periods are short, except for cases of distant metastasis, salvage surgery has resulted in disease-free survival with preserved quality of life in our cases. Because endoscopic resection of NMM may vary by concept, e.g., en bloc resection or piecemeal resection, and by tumor location, future large-scale and detailed studies are required to evaluate the benefits of endoscopic resection of NMM.

**Figure 4:**
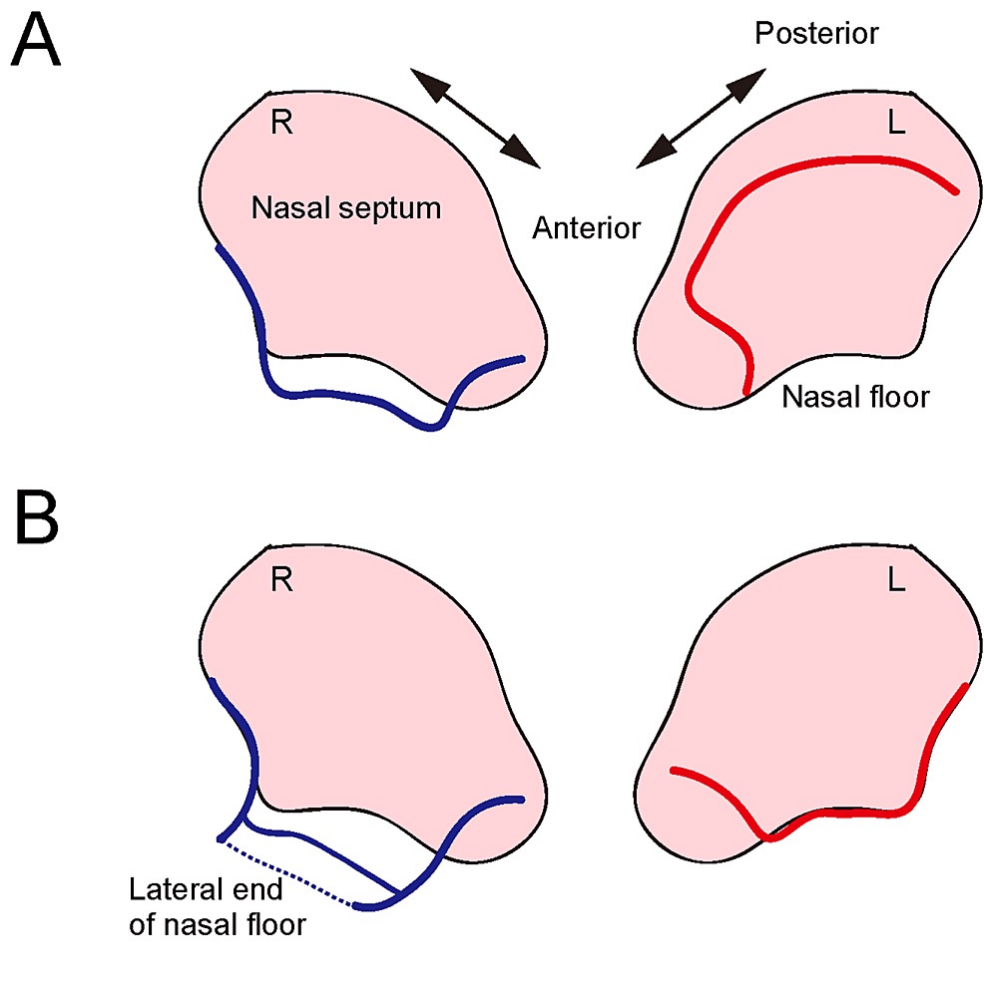
Incisions on both septal mucoperiosteum. A: Incisions on both septal mucoperiosteum of transseptal access and crossing multiple incisions (TACMI) (right intact side, blue line; left tumor side, red line). B: Incisions on both septal mucoperiosteum per our method. The right mucosal flap is raised thoroughly to the lateral end of the nasal floor.

In the present cases, we performed an en bloc resection of NMM of the inferior turbinate using temporal transseptal access, based on previous reports [8, 9] with modifications. In the original report, Omura K et al. proposed the transseptal access with crossing multiple incisions (TACMI) technique, allowing transposition of the tumor to the intact lateral nasal cavity without a post-operative septal perforation [9]. A septoplasty is performed to remove the excess bone and cartilage, and anterior vertical and superior horizontal incision lines (on the tumor side) and anterior vertical, posterior vertical, and inferior horizontal incision lines (on the intact side) are made. The risk of subsequent septal perforation is minimal because the two sides' incisions do not cross each other [8, 9] (Figure 4A). Although this technique provides an excellent surgical field of view and working space, one requires adequate surgical skills to make an appropriate mucosal incision on the tumor side, which is usually occupied by the tumor, and bilateral mucosal sutures along the inferior and superior horizontal incisions for septal reconstruction.

We raised a septal mucosal flap, with or without an inferior horizontal incision, on the intact side, similar to that in the TACMI technique (Figure 4B). However, in contrast to that in the original technique, we made an identical septal mucosal flap on the tumor side with a horizontal incision. Both mucosal flaps could be easily moved to the lateral side of the intact nasal cavity. The transseptal access made by this technique is sufficient to move the nasal cavity-occupying tumor to the contralateral side. This creates a working space to perform "two nostrils-three or four hands" endoscopic surgery, to visualize a deeper space, and to achieve negative resection margins. After successful tumor resection, flaps can be returned to the medial position without the need for bilateral mucosal sutures along the superior horizontal incisions for septal reconstruction. Although we used fibrin glue to attach the flaps to each other and the nasal floor, this might be unnecessary when the vertical length of the intact side's flap is undamaged and can reach the medial part of the nasal floor. This transseptal access technique has some limitations, including increased operation time and the risk of septal perforation. However, these limitations can be overcome with increased experience. The balance between operating time and surgical invasion should be considered in each case.

## Conclusions

In conclusion, en bloc resection of NMM using temporal transseptal access and endoscopic surgery is a feasible method for treating resectable NMM localized in the inferior turbinate. Although there are new options, such as proton beam therapy and targeted therapy for treating NMM, elderly patients may not be eligible for these treatments due to their social and physical limitations. Nevertheless, our method is easy but effective and is worth considering to avoid open craniofacial resections.
